# Vibration Perception Threshold and Related Factors for Balance Assessment in Patients with Type 2 Diabetes Mellitus

**DOI:** 10.3390/ijerph18116046

**Published:** 2021-06-04

**Authors:** Jisang Jung, Min-Gyu Kim, Youn-Joo Kang, Kyungwan Min, Kyung-Ah Han, Hyoseon Choi

**Affiliations:** 1 Department of Rehabilitation Medicine, Nowon Eulji Medical Center, Eulji University School of Medicine, 68 Hangeulbiseok-ro, Nowon-gu, Seoul 01830, Korea; q614@naver.com (J.J.); kmg5201@naver.com (M.-G.K.); md52516@hanmail.net (Y.-J.K.); 2 Diabetes Center, Department of Internal Medicine, Nowon Eulji Medical Center, Eulji University School of Medicine, 68 Hangeulbiseok-ro, Nowon-gu, Seoul 01830, Korea; minyungwa@gmail.com (K.M.); hka114@gmail.com (K.-A.H.)

**Keywords:** diabetes mellitus, vibration perception threshold, neuropathy, balance, posturography, fear of falling

## Abstract

Diabetic peripheral neuropathy (DPN) is a common complication of type 2 diabetes mellitus (DM). DPN causes a decrease in proprioception, which could reduce balance ability. We investigated the association of impaired vibration sense, based on vibration perception threshold (VPT), with assessments of balance and other factors affecting balance impairment and fear of falling in patients with type 2 DM. Sixty-three patients with DM aged >50 years were categorized as having normal vibration sense (NVS; *n* = 34) or impaired vibration sense (IVS; *n* = 29) according to a VPT value of 8.9 μm. The following parameters were evaluated for all patients: postural steadiness through the fall index using posturography, functional balance through the Berg Balance Scale (BBS), the Timed Up and Go test (TUG), and fear of falling through the Falls Efficacy Scale-International (FES-I). The IVS group showed a significantly greater balance impairment in fall index, BBS, and TUG, as well as greater fear of falling on the FES-I than the NVS group. The linear regression analysis showed that the fall index was associated only with the VPT, whereas BBS, TUG, and FES-I were associated with the VPT, age, and/or lower extremity muscle strength. VPT, age, and/or muscle strength were identified as predictors of balance and fear of falling in patients with type 2 DM. Therefore, along with age and lower extremity strength, the VPT can be useful for balance assessment in patients with type 2 DM.

## 1. Introduction

Diabetes is a serious and chronic condition that negatively affects human life and burdens the health care system. The global prevalence of type 2 diabetes mellitus (DM) is increasing annually and was estimated at 6059 cases per 100,000 in 2017, and the number is predicted to rise to 7079 cases per 100,000 by 2030 [1]. The global diabetes-related health expenditure was estimated to increase to USD 760.3 billion in 2019, and is predicted to increase to USD 845.0 billion by 2045 [2]. Diabetes can cause serious damage to the heart and blood vessels, eyes, kidneys, nerves, and teeth, which can lead to serious health problems [3]. Diabetic peripheral neuropathy (DPN) is one of the most common complications of type 2 DM [4,5]. Adults with type 2 DM have about 40% prevalence of DPN [6]. DPN is associated with foot ulceration, amputation [7], and balance impairment in patients with type 2 DM [8].

The proprioceptive feedback of the lower extremities, in combination with visual and vestibular senses, provides the main sensory information to maintain and control posture. DPN causes a decrease in proprioception, such as a sense of position, decrease in the sense of vibration, loss of somatosensory feedback, and inappropriate motor response, which can reduce the ability to control balance [9,10]. In fact, patients with DPN are at a high risk of falling [11,12,13,14,15,16]. Falling has destructive consequences, including a decline in mobility, activity avoidance, institutionalization, mortality, and higher social and economic burden [8,17].

DPN can be assessed in several ways using different types of symptom scores and several kinds of measurements [18]. The vibration perception threshold (VPT) with a quantitative sensory testing (QST) approach was demonstrated to be a useful screening tool in many studies [19,20,21,22]. Because vibration sense is often impaired in large myelinated nerve fiber deficits such as in DPN, the VPT is widely used for screening for large fiber function in DM [23,24]. High VPT values are associated with DPN symptoms and predictive of serious complications, such as foot ulcers and amputation [25]. VPT is painless [26] and practical to implement in routine clinical care. Information on the VPT and predicting balance impairment would be helpful for identifying patients who need balance assessments and providing patient education to prevent falls in outpatient clinics [27]. Although several studies have evaluated balancing ability in patients with DPN, the assessment tools for DPN are very diverse across studies [10,13,16,20,28,29]. Few studies have comprehensively evaluated the association between the VPT and various balance assessment methods.

Identifying factors associated with balance impairment is another essential part of fall risk assessments of patients with type 2 DM. Although previous studies have reported that DPN is associated with an impairment of balancing ability [11,12,13,14,15,16], it can be expected that several factors cause balance impairment in diabetic patients either alone or in combination with DPN. However, studies on factors related to balance and fall risk in patients with type 2 DM are still insufficient, and the results are unclear [15]. Functional balance plays an important role in performing many complicated tasks during daily life [14,16]. Since the body tries to find additional ways to reduce postural instability at the functional level [14,15], related factors may differ for postural steadiness and functional balance. In addition to balance impairment, psychological factors, such as a fear of falling, with or without a history of falling, are risk factors for falls. A fear of falling is a psychological obstacle to the performance of physical activities and is predictive of future falls [30]. Both physical and psychological approaches will enable more focused fall risk assessment. Thus, it is necessary to evaluate factors related to balance ability through both physical and psychological assessments, including postural steadiness, functional balance, and the fear of falling.

The objective of this study was: (1) to investigate the association between VPT and various balance measures, including postural steadiness, functional balance, and fear of falling, and (2) to identify significant factors affecting balance and fear of falling in patients with type 2 DM.

## 2. Materials and Methods

### 2.1. Subjects

In this cross-sectional study, a total of 63 patients with type 2 DM were recruited as subjects among patients who visited the diabetes clinic in the Department of Endocrinology and Diabetes of the University Hospital for the purpose of controlling type 2 DM. All subjects were over 50 years of age, medically stable, understood the instructions (Mini Mental State Examination score >24), and were able to stand independently. Subjects were excluded if they had musculoskeletal disorders (e.g., spinal radiculopathy, foot ulcer, lower limb amputation), rheumatic disease, surgery (e.g., spinal surgery, replacement of lower limb joint), history of trauma of the lower extremity that could impair balance ability and lower extremity function, neurological disorders (e.g., stroke, Parkinson’s disease, multiple sclerosis, spinal radiculopathy), visual impairment, or any other vestibular disease. All subjects provided written informed consent, and this study was approved by the Institutional Review Board of our hospital (EMCS 2020-04-023).

### 2.2. Vibration Perception Threshold (VPT) Assessment

The VPT was measured using a vibratory sensory analyzer VSA-3000 (Medoc Ltd., Ramat Yishai, Israel), a quantitative sensory testing (QST) computerized device. The examiner, who was not involved in administering the balance outcome measures, explained VPT test methods to the patients. Before the test trial, three practice trials were conducted to allow the examiner to explain the procedure and to help the patient become acquainted with the test. The patients were asked to sit on a chair. The VPT was assessed at four sites of the bare testing sole (big toe, 1st metatarsal, 5th metatarsal, and heel) [31] by delivering a 100 Hz vibration stimulus. The method of limits was used for the vibratory thresholds [32]. The intensity of the stimulus was increased at a rate of 0.8 μm/s from the baseline (0 μm). As soon as the patient detected the stimulus, a button was pressed to stop the machine from delivering it, and thereafter the next trial was initiated from the baseline. The average result of three trials was considered as the VPT for each foot site. Finally, the VPT value was taken as the average VPT from the eight site measurements. Impaired vibration sense was detected using the threshold value of VPT >8.9 μm, which was reported to be the best cut-off value for detecting DPN [20]. Subjects were classified into normal vibration sense (NVS) and impaired vibration sense (IVS) groups, based on the VPT cut-off value of 8.9 μm.

### 2.3. Nerve Conduction Study (NCS) Assessment

The nerve conduction study (NCS) was performed to assess DPN severity by the physician experienced in the electrodiagnostic study. The composite score was calculated to confirm the significant difference in DPN severity between the two groups based on VPT, and the correlation between VPT value and composite score. The composite score consisted of peroneal motor distal latency, peroneal motor amplitude, peroneal motor conduction velocity, tibial motor distal latency, and sural sensory amplitude. The peroneal motor nerve was stimulated at the ankle and fibular head, with recording over the extensor digitorum brevis muscle. The conduction velocity was measured between the ankle and fibular head. The tibial motor nerve was stimulated posterior to the medial malleolus, with recording over the abductor hallucis muscle. The sural nerve was stimulated at 14 cm proximal to the lateral malleolus in the posterior calf line, with recording posterior to the lateral malleolus. Each component was transformed to an abnormal percentile relative to the distribution of the values of normal NCS (<95th = 0; ≥95th–99th = 1; ≥99th–99.9th = 2; ≥99.9th = 3). The points of the five components were totaled, divided by the number of attributes with available values, and multiplied by five [33].

### 2.4. Postural Steadiness Assessment

Postural steadiness was evaluated by static posturography using the Tetrax^®^ (Sunlight Medical Ltd., Ramat Gan, Israel) with the supervision of a physical therapist. The device has four individual plates (A-B-C-D, A: left heel; B: left toes; C: right heel; D: right toes) for measuring the vertical pressure fluctuations with a single axis load cell, which is one of the load sensor types using the strain gauge [34]. The vertical pressure distributed across each force plate was measured while standing in an upright position for 32 s. The data of vertical pressure fluctuations were computerized and analyzed by the Tetrax^®^ software to obtain the fall risk index, stability index (ST), Fourier index (FI), weight distribution index (WDI), and synchronization index. The patients stood in the center of four force plates and were instructed to maintain eight different postures, such as head rotation, upward, and downward, with eyes open, eyes closed, and on unstable surfaces. In this study, the postural steadiness was estimated with reference to the fall risk index, ST, and FI for a medium to high frequency of 0.5–1 Hz [11,34,35,36,37].

Fall risk index: The fall risk index is globally calculated using the data of ST, FI, WDI, and synchronization index, considering the oscillation velocities computed by the Tetrax^®^ program [34,36,37]. It is numerically expressed from 0 to 100. A value of 0–36 indicates low risk, 37–58 indicates moderate risk, and 59–100 indicates high risk. A higher fall risk index indicates a greater risk of falling [34,35,36,37].

Stability index (ST): The ST indicates the patient’s overall stability and ability to control postural change. The amount of postural sway is analyzed using the square root of the sum of the squared differences between consecutive pressure signals. Then, it is divided by the patient’s body weight [34,35,37]. A higher index score indicates more postural instability [34,35,36,37].

Fourier index (FI): The FI is a regression analysis of the intensity of postural sway mathematically calculated using a Fourier transformation. It represents the frequency of postural sway within a variable spectrum between 0.01 and 3.0 Hz. Fourier power values are compared with the regression curve calculated by the software, evaluating the difference of coefficient between a graph of measured data and theoretically ideal regression [37]. The FI value for medium to high frequency of 0.5–1 Hz (F 5–6) is high when the patients present with somatosensory dysfunction of the lower extremities and spine [34,38]. A higher index score for frequency represents greater postural instability [35,36,37].

### 2.5. Functional Balance Assessment

The functional balance assessments were conducted by a physical therapist with 30 min of rest period after a postural steadiness assessment. The general instruction of functional balance tests was explained before the actual examination.

Berg Balance Scale (BBS): The BBS is a tool for evaluating the functional balance required for movements related to actions encountered in daily life. It is composed of 14 tests, with each test scored from 0 to 4 points. The maximum score is 56 points, and a higher score reflects better functional balance [14,39,40].

Timed up and go (TUG) test: The TUG test was performed 30 min after the evaluation of BBS. The TUG test is a dynamic functional balance test, which measures the time it takes to stand up from the chair, turn around at a turning point at a 3 m distance at ordinary walking speed, and sit back on the same chair. The TUG test is useful for assessing a person’s functional mobility and the risk of falls in community-dwelling older people. In older adults, if the TUG test requires more than 12 s, the risk of falls is high [41,42].

### 2.6. Fear of Falling Assessment

Falls Efficacy Scale-International (FES-I): The FES-I is a widely used self-report questionnaire tool for evaluating concerns about falls while carrying out routine activities. The FES-I is simple and easy to administer, and its score categories cover both easy and difficult physical and social activities inside and outside the house [43]. It is composed of 16 items that evaluate basic activities of daily living, with each item scored from 1 to 4 points. The total score ranges from 16 to 64 points, and a higher score reflects a greater fear of falling when performing daily activities [44,45].

### 2.7. Statistical Analysis

To confirm the differences in fall index, BBS, TUG, and FES-I between the two groups (NVS and IVS) in this exploratory study, the sample size was calculated based on effect size = 0.8 (the large effect by Cohen), alpha = 0.05, power = 0.8, by an independent sample t-test using G-power 3.1.2 (Heine Heinrich University, Dusseldorf, Germany). The minimum required sample size was 26 in each group. Therefore, it was intended to enroll more than 26 patients per group. The baseline characteristics were analyzed using an independent sample t-test for continuous variables and a chi-squared test for nominal variables. An independent sample t-test was performed to assess the difference in postural steadiness and functional balance capabilities between the two groups. A Pearson’s correlation analysis was used to assess the correlation between balance parameters and VPT. Finally, a stepwise multiple linear regression was used to evaluate factors that affect postural steadiness and functional balance, respectively. Independent variables included age, sex, height, weight, body mass index (BMI), DM duration, glycated hemoglobin levels (HbA1c), Medical Research Council (MRC) score, presence of hypertension, chronic kidney disease, retinopathy, and the value of the VPT. To determine the final regression model, a variable was added to or removed from the model according to the repeated stepwise method algorithm that included variables for which the *p*-value was <0.05, and removed variables for which the *p*-value was >0.1. All statistical analyses were performed using SPSS version 22.0 (IBM, Armonk, NY, USA). Statistical significance was set at *p* < 0.05.

## 3. Results

### 3.1. Patient Characteristics

Sixty-three subjects were recruited for this study and completed all the tests. Based on the VPT value of 8.9, the patients were classified into two groups: NVS (*n* = 34) and IVS (*n* = 29) groups. Demographic and clinical differences between the two groups are shown in Table 1. The IVS group was associated with higher VPT values and longer diabetes duration than the NVS group (*p* < 0.01). The composite score in the NCS was significantly higher in the IVS group than in the NVS group (*p* < 0.001). In the correlation analysis, there was a significant correlation between VPT and composite score (r = 0.673, *p* < 0.05). The differences in the other studied parameters (i.e., group, sex, age, height, weight, BMI, HbA1c, MRC score, hypertension, DM nephropathy, retinopathy, chronic heart disease, and chronic lung disease) were not significantly different between the two groups (*p* > 0.05). Regarding the MRC score, five patients had lower limb weakness in the IVS group even though they had no musculoskeletal or neurological disorders belonging to the exclusion criteria of this study.

### 3.2. Differences in Postural Steadiness, Functional Balance, and Fear of Falling between the NVS Group and IVS Group

Table 2 and Table 3 show a comparative analysis of the postural steadiness parameters: fall risk index, ST, and FI 5–6 for both groups using Tetrax^®^. The fall risk index in the IVS group was significantly higher than that in the NVS group (39.1 vs. 82.9%, *p* < 0.001; Table 2). The ST and FI 5–6 of the IVS group were significantly higher than those of the NVS group, regardless of postural conditions (*p* < 0.05, Table 3).

Table 4 shows a comparative analysis of functional balance parameters and the fear of falling in both groups. Categorization in the IVS group was associated with a decreased BBS (52.0 vs. 43.7 points, *p* < 0.001), with increased TUG test scores (8.8 vs. 12.0 s, *p* = 0.001) compared to the NVS group. The FES-I score was significantly higher in the IVS group than in the NVS group (20.2 vs. 26.0 points, *p* < 0.001).

### 3.3. Correlations between the VPT Value and Postural Steadiness, Functional Balance, and the Fear of Falling

Using Pearson’s correlation, the VPT value was strongly correlated with postural steadiness parameters of the fall risk index (r = 0.730, *p* < 0.001; Table 5). The VPT value was significantly correlated with the functional balance parameters of BBS (r = −0.680, *p* < 0.001) and TUG test (r = 0.417, *p* = 0.001; Table 5). The VPT value was also significantly correlated with the FES-I (r = 0.423, *p* = 0.001; Table 5).

### 3.4. Factors Contributing to Postural Steadiness, Functional Balance, and the Fear of Falling in Patients with Type 2 DM

A multivariate regression analysis was performed using the significant factors in univariate regression analysis to investigate the factors related to postural steadiness, functional balance, and fear of falling. The fall risk index was significantly associated only with VPT values after adjusting for covariate factors. Not shown in the table, VPT was the only significant predictor of the STs and FIs (F 5–6) for eight postural conditions.

Table 6 displays the final multivariate linear regression model for the fall risk index, BBS, TUG, and FES-I. In the multivariate analysis of fall risk index, a higher VPT was associated with a higher fall risk index with a beta coefficient (95% CI) of 2.472 (1.881–3.064), *p* < 0.001. In the multivariate analysis of BBS, a higher VPT and older age were associated with lower BBS with beta coefficients (95% CI) of −0.412 (−0.529–−0.294), *p* < 0.001; and −0.264 (−0.369–−0.160), *p* < 0.001, respectively. In the multivariate analysis of TUG, a higher VPT, older age, and lower MRC score were associated with a higher TUG with beta coefficients (95% CI) of 0.103 (0.017–0.188), *p* = 0.020; 0.209 (0.134–0.283), *p* < 0.001; and −1.848 (−3.133–−0.562), *p* = 0.006, respectively. In the multivariate analysis of FES-I, higher VPT, older age, and lower MRC score were associated with higher FES-I with beta coefficients (95% CI) of 0.209 (0.050–0.369), *p* = 0.011; 0.207 (0.067–0.347), *p* = 0.004; and −2.871 (−5.270–−0.472), *p* = 0.020, respectively.

## 4. Discussion

These results indicate that patients with DM with IVS, as indicated by the VPT, exhibited decreased postural steadiness and functional balance and increased fear of falling compared to those with NVS. The current study also suggests that the VPT value, as well as the presence of IVS, were significantly associated with postural steadiness, functional balance, and fear of falling. Diminished vibration sense contributes to balance deficits and increased fall risk [46,47]. In Hafström’s study [46], decreased vibration sense had negative effects on perceived and functional balance in relatively healthy older adults. In Bergin’s study [47], vibration perception was increased in the patients with peripheral neuropathies, and there was a significant correlation between vibration sense and body sway. The results of our study are consistent with the results of previous studies for diabetic patients, which suggested that DPN assessed with various kinds of measurements was associated with postural steadiness and functional balance [14,29,48,49,50].

To date, the NCS is the most common method for the evaluation of DPN. However, it is painful, expensive, requires specific training, and quantitative assessment of the severity of DPN is more difficult compared to the use of VPT [26]. The VPT test can compensate for these limitations of the NCS. The VPT test is practical for clinical settings because it is non-invasive, painless, faster, and easier to perform compared to the NCS [18]. In our study, the VPT value showed a strong correlation with the composite score of NCS reflecting DPN severity. As a higher VPT value is associated with balance and fear of falling, this convenient [19] and reliable [21] quantitative assessment may represent a useful addition to the standardized evaluation of patients with type 2 DM.

We identified differences in factors related to postural steadiness and functional balance capability in patients with type 2 DM. In terms of postural steadiness, computerized posturography using Tetrax^®^ showed that only the VPT value influenced postural steadiness. Emam et al. reported that poor glycemic control, as assessed by HbA1c, along with DPN diagnosed by Hoffman’s reflex and electromyography, influenced postural instability using computerized posturography [51]. Guy et al. reported that quantitative sensory measurement of DPN and age were correlated with postural instability [10]. Differences across studies in factors reported affecting postural steadiness may be attributed to differences in the tools used to measure postural steadiness. In our study, the VPT value was the only factor that affected postural steadiness and showed an important correlation with postural balance using Tetrax^®^ posturography assessment. This result suggests that in cases where it is necessary to evaluate the balance deficit exclusively due to DPN, postural steadiness assessment may be required.

The results of this study showed that the VPT score and age had a significant association with the BBS and TUG test scores, which act as indicators of functional balance. This finding is consistent with previous studies that evaluated DPN by other methods [29,52]. Bogdan et al. reported that the severity of DPN as quantified using the Michigan Neuropathy Screening Instrument (MNSI), age, and depression symptoms were associated with impaired balance [29]. Further, Renata et al. reported that advanced age, absence of proprioceptive sense assessed by a 128 Hz tuning fork, disability, and absence of step strategy were associated with abnormal balance and mobility in elderly patients with type 2 DM [52]. Although there is a debate over whether the strength of the lower extremities affects balance ability in older people [53,54,55], lower extremity muscle strength of diabetic patients also affected the TUG test results in our study. The loss of muscle strength in these patients may have a significant impact on TUG test results, not on BBS. It can be speculated that the TUG test is affected by lower extremity muscle strength and the mobility aspect to a greater degree than the BBS tool. Moreover, the fall risk assessed by BBS may be underestimated due to a ceiling effect in patients with relatively higher levels of physical performance enrolled in this study.

It was identified that muscle strength is a factor that influences functional balance in patients with type 2 DM in this study. The muscle strength is related to the severity of DPN and may be caused by neurogenic atrophy due to axonal degeneration of the motor fibers [56]. The muscle strength was an independent predictor of functional balance measured by the TUG test after adjusting for other covariate factors. These results suggest that both VPT evaluation and lower extremity muscle strength should be regularly monitored for balance assessment in patients with type 2 DM.

The patients with IVS had a greater fear of falling than patients with NVS. This result is consistent with previous studies [29,57]. In Bogdan’s study [29], the FES-I score was significantly higher in the DPN group compared to the without DPN group based on MNSI. In Riandini‘s study [57], a fear of falling measured by FES-I showed a significant positive correlation with DPN severity quantified using MNSI. Our findings indicated that the VPT, age, and lower extremity strength had a statistically significant association with the fear of falling. With regard to the FES-I findings, patients with IVS may have a concern about inappropriate somatosensory feedback from the lower limbs due to DPN. Patients with DPN who have a history of falls tend to have a fear of falling and a cautious gait, as indicated by a decrease in walking speed, reduction in step length, and an increase in step width [58,59]. Especially in older patients with DPN, a high concern for falls may lead to changes in postural control strategies, such as rigid compensatory strategies [28]. However, an excessively rigid compensatory strategy can further increase the risk of falls. In addition, older patients with IVS who are afraid of falls may lose confidence, resulting in limitations of physical activity, physical frailty, and falls. This may lead to a vicious cycle that accelerates the progression of diabetes, which further increases the severity of DPN. Therefore, evaluation of VPT is necessary for providing proper interventions to restore confidence and prevent falls.

### Limitations

This study had some limitations. First, because the follow-up evaluation was not performed, which is a limitation of cross-sectional studies, we could not confirm any association between changes in the VPT and changes observed in balance. A prospective follow-up study is required in the future. Second, for evaluation of Tetrax^®^ posturography, patients with a relatively high level of functioning who could stand independently were enrolled in this study. However, it is meaningful that the VPT is useful for assessment of balance ability even in these patients who may not be suspected of poor balance in an outpatient setting. Using the advantage of VPT as a simple screening method, further research is needed in patients with type 2 DM with more severe disability and limited mobility.

## 5. Conclusions

This study demonstrated significant associations between VPT and several measures of balance ability in patients with type 2 DM. Postural steadiness was mainly affected by the VPT; however, age and/or muscle strength in the lower extremity were also related to functional balance and fear of falling. Therefore, along with age and lower extremity strength, the VPT might be useful for balance assessment, thus serving as a marker for fall risk in patients with type 2 DM.

## Figures and Tables

**Table 1:** Baseline characteristics of patients with type 2 DM.

	NVS (*n* = 34)	IVS (*n* = 29)	*p*-Value
Male sex, *n* (%)	14 (41.2%)	14 (48.3%)	0.572
Age, years	66.76 ± 8.85	70.00 ± 10.62	0.192
Height, m	1.59 ± 0.09	1.60 ± 0.11	0.819
Weight, kg	67.1 ± 13.4	68.6 ± 14.0	0.660
BMI, kg/m^2^	26.28 ± 3.98	26.65 ± 3.22	0.687
DM duration, years	14.88 ± 10.02	22.48 ± 9.53	0.003 *
HbA1c, %	10.6 ± 2.6	10.2 ± 2.6	0.526
MRC score	20.0 ± 0.0	19.2 ± 0.8	0.184
Hypertension, *n* (%)	27 (79.4%)	17 (58.6%)	0.073
DM nephropathy, *n* (%)	7 (20.6%)	10 (34.5%)	0.216
Retinopathy, *n* (%)	11 (32.4%)	12 (41.4%)	0.458
Chronic heart disease, *n* (%)	5 (14.7%)	3 (10.3%)	0.716
Chronic lung disease, *n* (%)	1 (2.9%)	0 (0%)	>0.999
VPT, mean, μm	5.01 ± 1.88	18.57 ± 7.76	<0.001 *
NCS, Composite score	3.54 ± 3.20	9.15 ± 3.53	<0.001 *

Data are presented as number (*n*) and percentage or mean ± standard deviation. * *p* < 0.05 (calculated through independent sample t-test). DM: diabetes mellitus; NVS: normal vibration sense; IVS: impaired vibration sense; BMI: body mass index; HbA1c: glycosylated hemoglobin; MRC: Medical Research Council; VPT: vibration perception threshold; NCS: nerve conduction study.

**Table 2:** Postural steadiness in patients with DM with NVS vs. IVS according to VPT value.

	NVS (*n* = 34)	IVS (*n* = 29)	*p*-Value
Fall risk index	39.1 ± 15.6	82.9 ± 23.7	<0.001 *

Data are presented as mean ± standard deviation. * *p* < 0.05 (calculated through independent sample t-test). VPT: vibration perception threshold; DM: diabetes mellitus; NVS: normal vibration sense; IVS: impaired vibration sense.

**Table 3:** Stability index and Fourier indices in eight positions in patients with DM with NVS vs. IVS according to VPT value.

Position	Stability Index			Fourier Index (0.5–1.0 Hz, F 5–6)		
	NVS (*n* = 34)	IVS (*n* = 29)	*p*-Value	NVS (*n* = 34)	IVS (*n* = 29)	*p*-Value
Eyes open	15.4 ± 3.5	21.5 ± 5.7	<0.001 *	3.1 ± 0.8	4.1 ± 1.2	<0.001 *
Eyes closed	29.0 ± 9.5	42.4 ± 20.1	0.002 *	5.7 ± 2.0	7.9 ± 3.7	0.006 *
Eyes open (Pillow)	18.4 ± 5.2	28.0 ± 9.1	<0.001 *	3.5 ± 1.2	5.0 ± 1.8	<0.001 *
Eyes closed (Pillow)	36.1 ± 14.3	48.5 ± 17.6	0.003 *	6.8 ± 3.1	8.8 ± 3.3	0.018 *
Head right	27.3 ± 9.7	39.1 ± 15.9	0.001 *	5.1 ± 1.9	7.1 ± 2.7	0.001 *
Head left	26.7 ± 8.9	40.2 ± 14.3	<0.001 *	5.1 ± 2.0	7.2 ± 2.9	0.001 *
Head up	28.3 ± 8.8	44.2 ± 16.1	<0.001 *	5.0 ± 1.4	8.1 ± 3.2	<0.001 *
Head down	28.2 ± 7.2	38.5 ± 13.0	<0.001 *	5.4 ± 1.6	6.8 ± 2.9	0.019 *

Data are presented as mean ± standard deviation. * *p* < 0.05 (calculated through independent sample t-test). VPT: vibration perception threshold; DM: diabetes mellitus; NVS: normal vibration sense; IVS: impaired vibration sense.

**Table 4:** Functional balance and fear of falling in patients with DM with NVS vs. IVS according to VPT values.

	NVS (*n* = 34)	IVS (*n* = 29)	*p*-Value
BBS (points)	52.0 ± 3.3	43.7 ± 5.9	<0.001 *
TUG (s)	8.8 ± 1.8	12.0 ± 4.9	0.001 *
FES-I (points)	20.2 ± 4.0	26.0 ± 7.0	<0.001 *

Data are presented as mean ± standard deviation. * *p* < 0.05 (calculated through independent sample t-test). VPT: vibration perception threshold score; NVS: normal vibration sense; IVS: impaired vibration sense; DM: diabetes mellitus; BBS: Berg Balance Scale; TUG: Timed Up and Go test; FES-1: Falls Efficacy Scale-International.

**Table 5:** Correlations between VPT and postural steadiness, functional balance, and fear of falling in patients with type 2 DM.

	Correlation Coefficient, r (95% CI)	*p*-Value
Fall risk index	0.730 (0.588–0.834)	<0.001 *
BBS	−0.680 (−0.817–−0.541)	<0.001 *
TUG	0.417 (0.196–0.661)	0.001 *
FES-I	0.423 (0.177–0.642)	0.001 *

* *p* < 0.05 (refers to the *p*-value of Pearson’s correlation coefficient). VPT: vibration perception threshold score; BBS: Berg Balance Scale; TUG: Timed Up and Go test; FES-1: Falls Efficacy Scale-International.

**Table 6:** Factors associated with postural steadiness, functional balance, and fear of falling in patients with type 2 DM.

	Independent Variable	B (95% CI)	Standardized B	*p*-Value
Fall risk index	Intercept	31.447 (23.058–39.837)		
	VPT score	2.472 (1.881–3.064)	0.730	<0.001 *
BBS	Intercept	70.849 (63.848–77.851)		
	VPT score	−0.412 (−0.529–−0.294)	−0.574	<0.001 *
	Age	−0.264 (−0.369–−0.160)	−0.414	<0.001 *
TUG	Intercept	31.646 (5.279–58.012)		
	VPT score	0.103 (0.017–0.188)	0.230	0.020 *
	Age	0.209 (0.134–0.283)	0.527	<0.001 *
	MRC score	−1.848 (−3.133–−0.562)	−0.268	0.006 *
FES-I	Intercept	63.491 (14.291–112.692)		
	VPT score	0.209 (0.050–0.369)	0.291	0.011 *
	Age	0.207 (0.067–0.347)	0.324	0.004 *
	MRC score	−2.871 (−5.270–−0.472)	−0.257	0.020 *

* *p* < 0.05. DM: diabetes mellitus; BBS: Berg Balance Scale; TUG: Timed Up and Go test; FES-1: Falls Efficacy Scale-International; VPT: vibration perception threshold; MRC: Medical Research Council.

## Data Availability

The data presented in this study are available on request from the corresponding author. The data are not publicly available because they represent information that could compromise the privacy of the study participants.
